# Human Dermal Decellularized ECM Hydrogels as Scaffolds for 3D In Vitro Skin Aging Models

**DOI:** 10.3390/ijms25074020

**Published:** 2024-04-04

**Authors:** Estibaliz Fernandez-Carro, Ana Rosa Remacha, Irene Orera, Giuseppe Lattanzio, Alberto Garcia-Barrios, Jesús del Barrio, Clara Alcaine, Jesús Ciriza

**Affiliations:** 1 Tissue Microenvironment (TME) Lab, Aragón Institute of Engineering Research (I3A), University of Zaragoza, C/Mariano Esquillor s/n, 500018 Zaragoza, Spain; 808895@unizar.es (E.F.-C.); necaila@unizar.es (C.A.); 2 Institute for Health Research Aragón (IIS Aragón), Avda. San Juan Bosco, 13, 50009 Zaragoza, Spain; 3 Proteomics Research Core Facility, Instituto Aragonés de Ciencias de la Salud (IACS), 50009 Zaragoza, Spain; iorera.iacs@aragon.es (I.O.);; 4 Department of Anatomy and Histology, Faculty of Medicine, University of Zaragoza, 50009 Zaragoza, Spain; 5 Departamento de Química Orgánica, Instituto de Nanociencia y Materiales de Aragón (INMA), CSIC-Universidad de Zaragoza, 50009 Zaragoza, Spain; jdb529@unizar.es; 6 Centro de Investigación Biomédica en Red en Bioingeniería, Biomateriales y Nanomedicina (CIBER-BBN), 28029 Madrid, Spain

**Keywords:** biomaterials, decellularization, human dermal extracellular matrix hydrogels, skin aging-on-a-chip, microfluidics

## Abstract

Biomaterials play an important role in the development of advancing three dimensional (3D) in vitro skin models, providing valuable insights for drug testing and tissue-specific modeling. Commercial materials, such as collagen, fibrin or alginate, have been widely used in skin modeling. However, they do not adequately represent the molecular complexity of skin components. On this regard, the development of novel biomaterials that represent the complexity of tissues is becoming more important in the design of advanced models. In this study, we have obtained aged human decellularized dermal extracellular matrix (dECM) hydrogels extracted from cadaveric human skin and demonstrated their potential as scaffold for advanced skin models. These dECM hydrogels effectively reproduce the complex fibrillar structure of other common scaffolds, exhibiting similar mechanical properties, while preserving the molecular composition of the native dermis. It is worth noting that fibroblasts embedded within human dECM hydrogels exhibit a behavior more representative of natural skin compared to commercial collagen hydrogels, where uncontrolled cell proliferation leads to material shrinkage. The described human dECM hydrogel is able to be used as scaffold for dermal fibroblasts in a skin aging-on-a-chip model. These results demonstrate that dECM hydrogels preserve essential components of the native human dermis making them a suitable option for the development of 3D skin aging models that accurately represent the cellular microenvironment, improving existing in vitro skin models and allowing for more reliable results in dermatopathological studies.

## 1. Introduction

The skin is the largest organ in the human body, involved in essential processes of the body, such as a barrier function against the external environment [1], the main goal of human skin. However, skin is more than a physical barrier, with several cells distributed in three layers, orchestrating functions, such as the temperature and pH control, elasticity maintenance or vitamin D production [2]. The epidermis, the outermost layer responsible for the first line of defense against external agents and participating in substance exchange with the external environment, is composed of keratinocytes, melanocytes, immune cells, and surface microbiota [3,4]. The middle layer, a connective tissue with an embedded fibroblast called dermis, provides most of the skin’s mechanical properties through its dermal extracellular matrix (dECM), rich in collagens, elastic fibers, glycosaminoglycans (GAGs), growth factors, proteoglycans, and glycoproteins. Finally, the hypodermis, the innermost layer, is responsible for thermoregulation, energy storage, and hormone conversion [5,6]. 

In recent years, tissue engineering has sought to develop new in vitro human skin equivalents for pharmacological, cosmetic, and toxicological studies, due to ethical concerns associated with animal experimentation and the implementation of the EU Cosmetic Regulation 1223/2009 prohibiting animal testing for cosmetic purposes [6]. However, inherent differences between human and animal physiology have challenged new skin models in the translation of results. Thus, the straightforward commonly used 2D skin cultures do not fully represent the complexity of native tissue, leading to alterations in cell morphology and behavior, with 3D models providing closer skin physiological representation, facilitating cell–cell and cell–matrix interactions [7]. In 3D models, synthetic materials such as collagen, fibrin or alginate are selected as a fibroblast-embedding scaffold to represent the dermis, with keratinocytes seeded on top to represent the epidermis. However, scaffold contraction [8], degradation [4], dermal fibroblast uncontrolled growth [9,10], and absence of native components from human dECM [6,7] are common limitations to faithfully reproduce the human dermis. 

Most reported 3D skin models’ studies generate scaffolds with type I collagen [6,7]. But human dECM is not solely composed of type I collagen, with a 3D network predominantly composed of various types of collagens, such as a collagen I, III or V, and elastic fibers, such as elastin, crucial for skin resilience and elasticity [11], or fibrilin-1 and -2, structural components that control the bioavailability of growth factors, also providing elasticity [12]. Thus, the fibrillar collagen type I is the most abundant in adult skin, with around 70–90% of collagen, participates in the dermal structure and integrity [13]. The fibrillar collagen type III, most abundant during prenatal development and around 10–21% in adult aged skin, also participates in dermal structure and integrity, providing mechanical properties such as tensility, flexibility, and softness [13,14,15]. The fibrillar collagen type V, around 2–8% of skin collagen, inserts and stabilizes type I collagen, contributing to epidermal cells migration [13,14,16]. Other minor collagen types of components in dermal ECM are the non-fibrillar collagen type VI, responsible for resistance to tensile stress and structural function [14,17], the non-fibrillar collagen type VII, stabilizing the dermal–epidermal junction (DEJ) [16], and the fibril-associated collagens with interrupted triple helices, type XII and XIV, that assemble and stabilize collagen fibrils [14,18]. 

Glycosaminoglycans (GAGs) also play a crucial role in the complex 3D framework [17], with hyaluronan responsible for skin hydration and osmotic balance [19], that in combination with dermatan sulfate, the most abundant GAG [20], participates in wound healing. Other components not reflected in synthetic materials reproducing the dermis are glycoproteins, similar to fibronectin connecting cells with collagens and providing attachment points and structural function [14], periostin, highly expressed in developing skins and in adults wound healing [21], or proteoglycans, similar to decorin binding to collagens and fibronectin providing structural function [22], or dermatopontin, providing elasticity, tensile strength, and collagen fibrillogenesis [23]. All these dermal components should be reflected in 3D skin aging model scaffolds to faithfully reproduce the natural dermal microenvironment if reliable preclinical evaluations must be provided.

Currently, reported studies with ECM hydrogels from diverse tissues have shown the preservation of native components [24]. However, reported dermal ECM hydrogels are extracted mainly from porcine skin [25,26], that exhibits similarities to human skin [27], but do not reproduce a completely adult human dermis since different preserved proteins have been shown between adult human and porcine ECM after decellularization, with different cell behaviors after exposure to matrices [28]. Therefore, dECM extracted from adult human samples can provide a more realistic microenvironment in equivalent skin aging models. The current manuscript describes the extraction and characterization of adult human dECM hydrogels that hold great promise as a powerful biomaterial to simulate more realistically adult human skin, providing more reliable future results.

## 2. Results and Discussion

### 2.1. Sol-Gel Transition of Adult Human Dermal Extracellular Matrix (ECM) at Physiologic Temperatures

**Figure 1:**
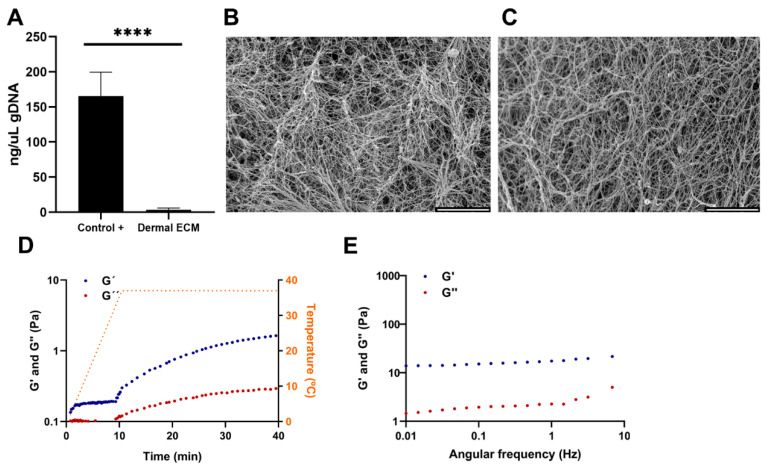
(**A**) Quantification of gDNA in human dECM. SEM images of (**B**) collagen and (**C**) human dECM hydrogels. Human dECM hydrogel (**D**) gelification kinetic and (**E**) frequency sweep. Note: Scale bar: 5 µm. G’: storage modulus. G”: loss modulus. Note: **** *p* < 0.0001.

Human skin is readily available in large quantities from adult cadaveric frozen tissues. There are few publications that compare the effect of preserving skin tissues by embalming and fresh freezing. It is known that the presence of boric acid in embalming solutions such as Thiel’s fluid has corrosive effects on proteins [29]. Furthermore, the preservation technique impacts the content and distribution of metabolites [30], with several chemicals used in embalming, such as phenol that can inhibit the activity of the required enzymes for the ECM extraction procedure [31,32]. Therefore, instead of selecting embalmed adult human skin tissues, frozen tissues were chosen and the human dECM digest was prepared from cadaveric adult human skin frozen samples by modifying previously described protocols [25]. The protocol consisted of decellularization and lyophilization, providing a sponge-like solid material (Figure S1A), that was further pulverized and digested (Figure S1B), to successfully generate human dECM hydrogels at a concentration of 4 mg/mL after neutralization (Figure S1C). The hydrogels were formed at a neutral pH, allowing for the subsequent embedding and culture of cells within the hydrogels. Decellularization involved the removal of cells from the tissue, avoiding degradation of ECM components [33], and enzymatic digestion to solubilize the ECM. The decellularization process significantly reduced the amount of DNA present in the human dECM digest to 14.68 ± 2 ng/µL (Figure 1A), that corresponds to an average of 45 ng DNA per mg dry mass, evidenced by the lack of DNA bands in agarose gel electrophoresis and haematoxylin-eosin stain where no cell nuclei were observed (Figure S1D,E). Since less than 50 ng of DNA per mg of dry mass avoids adverse cell and host responses and cytotoxic effect [33,34], we can confirm that the followed protocol is efficient in decellularizing human dermis. Decellularization processes usually involve tissue immersion in detergents with mechanical agitation, effectively dissociating DNA, and other cellular contents, but aggressively affecting 3D structure. Therefore, an optimal balance between cell removal and microarchitecture preservation must be set up [35]. Thus, electron microscopy images of human dECM hydrogels showed a fibrillar structure with randomly oriented fibers, similar to the collagen type I hydrogels (Figure 1B,C), with 3D porous that could allocate dermal fibroblasts or other cell types if desired, similar to collagen type I hydrogels porous.

Adult native skin exhibits an anisotropic and viscoelastic behavior [36], similar to most of the soft biological tissues. Various studies using different mechanical techniques have successfully characterized such a behavior in the skin, being dermis the main component that imparts this behavior to the tissue due to its fibrous network. The different types of collagens and elastic fibers, such as elastin, have great mechanical importance in this network [36,37,38,39,40,41]. Our sample preparation procedure was designed to simulate hydrogel gelation [25,42]. In such a procedure, a heating step from 0 to 37 °C over a period of 10 min triggers the gelation of the neutralized samples. In our samples, after reaching 37 °C, we observed that the human dECM hydrogels storage modulus (G’) and loss modulus (G”) increased with time and eventually reached a plateau, indicating that there is a transition from a fluid initial state to a structured and stable gelled state of the hydrogel, due to an internal cross-linking between proteins that provides an increase in stiffness (Figure 1D). According to the oscillatory frequency sweep measurements, values of the storage modulus were higher than the loss modulus values for all the frequency studies, indicating that human dECM hydrogels exhibit a predominantly elastic behavior in response to deformation (Figure 1E). In addition, the oscillatory amplitude sweep measurement revealed that the hydrogel’s stiffness and resistance increased with a greater applied deformation, thus exhibiting strain-stiffening behavior (Figure S2A), which has also been observed in other natural tissues and commercial collagen hydrogels [42]. 

### 2.2. Human Dermal Extracellular Matrix (ECM) Hydrogels Preserve Components Present in the Adult Native Tissue

**Figure 2:**
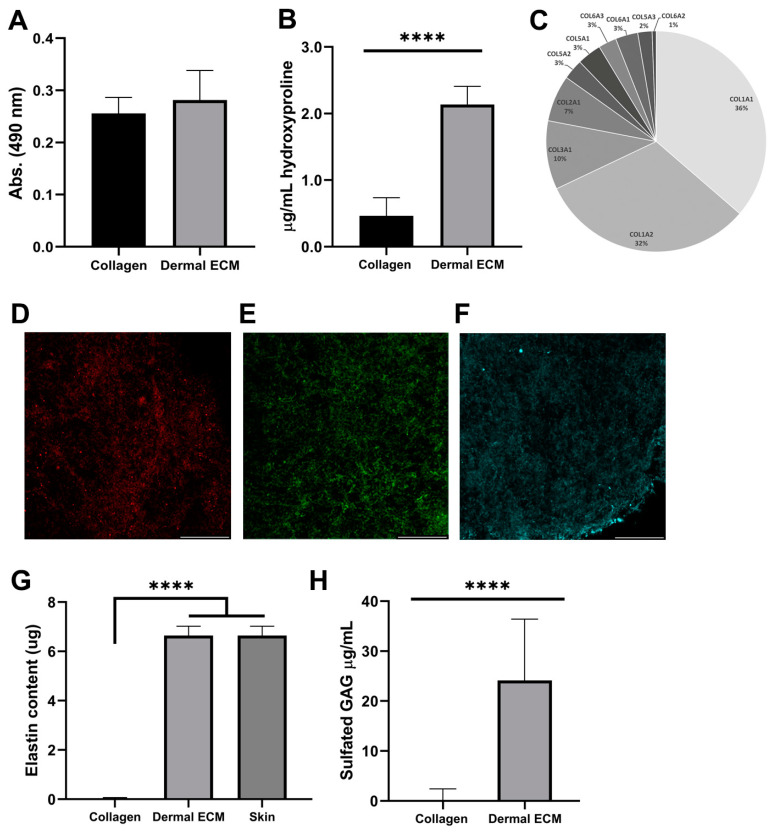
Human dECM (**A**) lipid and (**B**) hydroxyproline content. (**C**) Collagen types identification by mass spectrometry. Immunofluorescence of (**D**) collagen type I, (**E**) collagen type III, and (**F**) fibronectin. Human dECM (**G**) elastin and (**H**) sulfated GAG content. Note: Scale bar: 50 µm. **** *p* < 0.0001 compared with control.

In contrast to hypodermis, dermis is not a lipid-rich tissue. However, since the presence of lipid hinders the decellularization of the matrix and the subsequent gelation [43], the proper separation of dermis from the subcutaneous layer is required to avoid the presence of lipids. We quantified the lipid content in the human dECM hydrogel after Oil Red staining and further extraction, confirming a similar lipid amount in dECM (0.28 ± 0.06 absorbance units) and collagen hydrogels (0.26 ± 0.03 absorbance units) (Figure 2A), indicating a correct separation of dermis from the subcutaneous layer and no further inhibition by lipids in human dECM gelation.

Since decellularization can also affect the protein composition of human dECM [17], we studied the protein composition of human dECM by liquid chromatography mass spectrometry to determine if it was preserved from the adult native tissue. Collagens were the most abundant proteins in human dECM, along with other proteins, such as keratin types or structural proteins (Table S1). Therefore, we quantified the hydroxyproline concentration from human dECM hydrogels, as a major non-proteogenic amino acid of collagen, stabilizing its helical structure, and as an indicator of total collagen content. Thus, the hydroxyproline content of human dECM hydrogels (2.1 ± 0.3 µg/mL) was four-fold significantly higher than rat tail collagen type I hydrogels (0.5 ± 0.3 µg/mL) (Figure 2B), indicating the presence of other collagen types, such as type I, III or V [17]. Subsequently, we confirmed the presence of collagen type I and III, the most common collagens in the adult dermis and previously identified in mass spectrometry data (Figure 2C), by visualizing their expression under confocal microscopy (Figure 2D,E and Figure S2B). Collagen type III is the most abundant collagen during the prenatal period, while collagen type I shows a 5–6:1 (collagen I/collagen III) ratio in adults [16,44]. This ratio was confirmed by mass spectrometry, comparing the intrasample abundance of each protein, emPAI values, from different collagens. Along with other collagen types, they provide structure and tensile strength to the skin, also interacting with fibronectin that stabilizes collagen, and providing attachment sites for ECM–cell interaction [45]. We also confirmed fibronectin expression in the human dECM hydrogels under confocal microscopy (Figure 2F). The presence of dermatopontin, fibrillin-1 or actin was also detected by mass spectrometry in the human dECM (Table S1). The non-collagenous protein dermatopontin from dECM architecture is involved in collagen fibrillogenesis, and cell–cell adhesion [46,47], while fibrillin-1 confers elastic properties to dECM [48] and actin regulates collagen synthesis by fibroblasts [49]. To discard contamination from the epidermal layer during human dECM extraction, we also confirmed the lack of collagen type IV expression in human dECM hydrogel samples under confocal microscopy, previously undetected by a mass spectrophotometer (Figure 2C and Figure S2C and Table S1), characteristic of the basement membrane between the dermis and the epidermis, also known as the dermal–epidermal junction. This absence also assured the proper separation of dermal from the epidermal layer during the extraction procedure [50]. To complement the protein content characterization of human dECM hydrogels, we also quantified elastin preservation after detection by mass spectrometry (Table S1), since this low-expressed protein interacts with ECM fibers, allowing for the formation of a fibrous and viscoelastic network, and providing elasticity to the skin and resilience to restrain stretching [51]. Human dECM hydrogels successfully preserved elastin (6.64 ± 0.38 µg) up to concentrations from adult native skin (6.87 ± 0.481 µg) (Figure 2G), in contrast to non-human dECM hydrogels that have shown a decrease in elastin composition compared to the native tissue that has been reported [51]. 

Finally, we quantified the amount of sulfated glycosaminoglycans (sGAGs), such as chondroitin sulfate, heparan sulfate and dermatan sulfate, with roles in cellular signaling, skin hydration, tissue repair, infection clearance, fibrosis, and carcinogenesis [18]. Human dECM hydrogels exhibited significantly higher sGAGs concentration (24.2 ± 12.6 µg/mL) than commercial collagen hydrogels (0.6 ± 1.9 µg/mL) (Figure 2H), indicating that human dECM hydrogels can reproduce better the aforementioned native dermis roles in skin models. Altogether, our results demonstrate that human dermal ECM hydrogels preserve most of the components present in the adult native tissue, which is required to closely mimic human dermis in skin aging in vitro models.

### 2.3. Human dECM Hydrogels Are Biocompatible and Suitable for Use as Scaffolds in a Skin Aging-on-a-Chip Model

Human dECM allows for the generation of a scaffold in skin-on-a-chip models that represent a more realistic cellular microenvironment of the dermis, mimicking the physical and biochemical environments in vivo [52]. The use of a microfluidic system allows for improved spatial-temporal control and miniaturization, resulting in benefits such as high-performance, low cost, and low reagent consumption. Additionally, this system provides the possibility of fine-tuning conditions, integration of biosensing, and automation [53,54]. 

**Figure 3:**
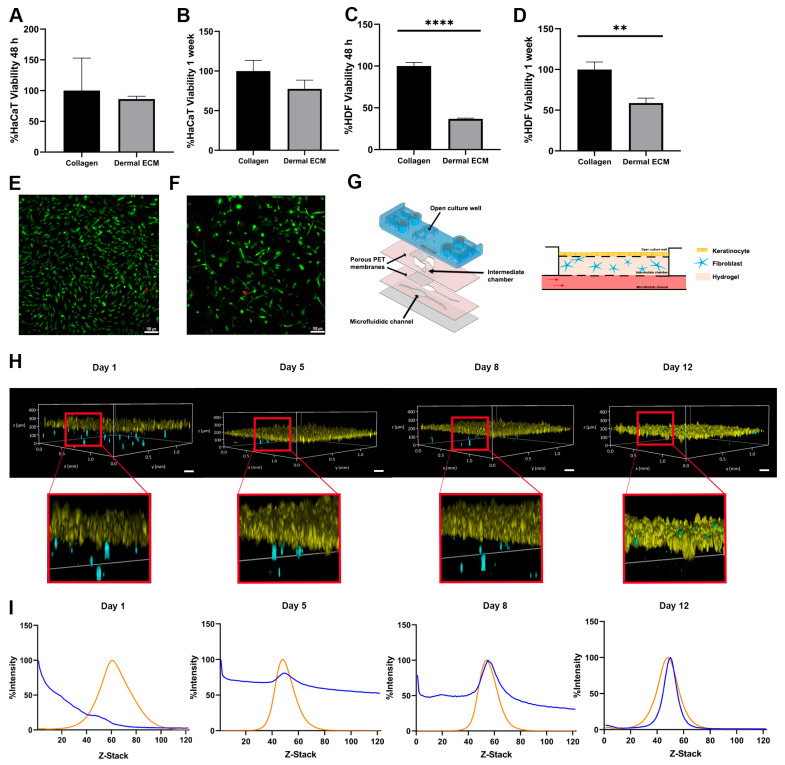
Metabolic activity of (**A**,**B**) keratinocytes seeded on human dECM hydrogel surfaces and (**C**,**D**) fibroblast embedded within human dECM hydrogels, compared with collagen type I hydrogels after 48 h and 1 week. Micrographs of fibroblast viability staining (green and red colors represent alive cells and death cells) within (**E**) collagen type I and (**F**) human dECM hydrogel. (**G**) Customized microfluidic devices based on COP and schematic representation of skin-on-a-chip model. (**H**) Keratinocytes (yellow) on top of human dECM hydrogels and fibroblast embedded in human dECM hydrogel (blue) tracking over time within a skin-on-a-chip device. (**I**) Analysis of cell migration quantifying the fluorescence intensity of fibroblasts (blue) embedded in human dECM hydrogel and keratinocytes on top of hydrogel (orange) over time within skin-on-a-chip device. Note: Scale bar: 100 µm. **** *p* < 0.0001,** *p* < 0.001.

Therefore, we determined its biocompatibility with the main cellular actors in skin, keratinocytes, and fibroblasts in an adult human dECM hydrogel. First, we quantified the metabolic activity of both cell types in contact with adult human dECM scaffolds. Thus, we embedded the dermal fibroblast within human dECM scaffolds, while keratinocytes were cultured over the hydrogels. After 48 h and one week of cell culture, keratinocytes did not show statistically significant metabolic activity differences with keratinocytes growth over commercial collagen hydrogels, indicating that biocompatibility was not affected by the components from the human dECM (Figure 3A,B). However, the metabolic activity from fibroblasts embedded within the hydrogels was significantly reduced up to approximately 50% (Figure 3C,D). Therefore, we studied fibroblast death by Live/Dead staining and further observation under fluorescent microscopy after one week of cell culture. Interestingly, no significant cell death was detected, with embedded fibroblasts maintaining similar morphology than the embedded fibroblast within collagen hydrogels (Figure 3E,F). These results suggest that the difference between embedded fibroblasts within human dECM and collagen may be attributed to an uncontrolled proliferation of fibroblasts from commercial collagen hydrogels, being human dECM capable of attenuating proliferation, consistent with data from metabolic activity assays. The wide variety of biomolecules present in human dECM helps to regulate fibroblast behavior, differentiation, and migration. Cell–cell and cell–dECM interactions and the presence of soluble factors and small molecules may control cell proliferation, modify the translation of cellular signals within the hydrogel, and create a scaffold that more realistically mimics the complex dermal microenvironment. In addition, the presence of sGAGs, such as the previously identified chondroitin sulphate (Table S1), may play a key role in controlling fibroblast proliferation due to their ability to sequester growth factors [53,55]. In fact, an excessive fibroblast proliferation would represent an altered situation, since an unbalance of ECM components leads to altered cell behavior [56], not being representative for adult natural healthy dermis. Moreover, high fibroblast densities provoke the shrinkage of the collagen hydrogels (Figure S2D) due to their contractile capacity, ruining the architecture of the 3D model, as shown by the calcein/propidium iodide staining fibroblast within collagen hydrogels. However, shrinkage is avoided when similar studies are performed with the same density embedded fibroblast within adult human dECM hydrogels. Therefore, a controlled growth of embedded fibroblast is essential to keep the hydrogel architecture, allowing for a long lifespan of the skin model [57,58], while closely representing the natural microenvironment of the adult skin.

Next, we aimed to monitor the embedded fibroblast growth and migration within the human dECM in a microfluidic skin model. We first designed a new microfluidic device based on cyclic olefin polymers (COPs) that allows for inserting the embedded fibroblast into one chamber in contact through a 1 μm porous polyethylene terephthalate membrane to a second open chamber where keratinocytes were grown (Figure 3G). Both fibroblasts and keratinocytes were fluorescently labeled and monitored over time. Embedded fibroblasts exhibited migration within the human dECM hydrogel, while the keratinocytes maintained a stable layer until day 12 (Figure 3H). In fact, the fibroblast migrated continuously to the upper part of both types of hydrogels over time, allocating close to the epidermal layer. However, most of the fibroblasts within adult human dECM hydrogels migrated progressively to the top of the hydrogel, with the majority of fibroblast in the dermal–epidermal junction at day 12 of culture, simulating the high fibroblasts density thin ECM layer of the papillary dermis [59], while fibroblasts within collagen type I hydrogels showed a more spread distribution over the hydrogel, even at day 12 of culture, as shown by the Z-stack fluorescence intensity analysis of the hydrogels over time (Figure 3I and Figure S2F). These results demonstrate that adult human dECM hydrogels reproduce closer to the dermis in a skin aging in vitro model than collagen type I hydrogels, reproducing the natural behavior of dermal fibroblast in terms of growth, migration, and distribution.

## 3. Materials and Methods

### 3.1. Decellularized Human Dermal Extracellular Matrix (ECM) Hydrogels

Full thickness skin samples were harvested from three human cadavers, avoiding fat collection from the hypodermis, and then stored at −80 °C until processing. All subjects were between 70 and 90 years of age. All the experimental procedures were performed in compliance with protocols approved by the institutional animal care and use committee of the University of Zaragoza (Permit Number: CEICA PI22/119). Human dermis ECM hydrogels were developed following previously published protocols in other species with some modifications [25]. Briefly, skin samples were thawed and washed with tap water. Samples were incubated with 3 units/mL of dispase II (Thermo Fisher Scientific, Carlsbad, CA, USA) for 2 h at 37 °C, next separating mechanically the dermis from the epidermis. Collected dermis was incubated for 6 h at 37 °C with 0.25% trypsin (Thermo Fisher Scientific) and washed with deionized water three times for 15 min each time. Next, the solution underwent an incubation for 10 h with 70% ethanol, followed by a treatment with 3% H_2_O_2_ for 15 min and washing twice with deionized water for 15 min each time. After washing, the sample was treated with 1% Triton X-100 in 0.26% EDTA/0.69% Tris for 6 h with a fresh change for an additional 16 h, again washing three times with deionized water for 15 min each, finally incubating in 0.1% peracetic acid/4% ethanol for 2 h and washing twice with phosphate buffered saline (PBS) (Lonza, Walkersville, MD, USA) followed by two extra washing steps with deionized water. All the procedure was carried out under constant agitation (300 rpm). At this time, the samples were lyophilized (LyoQuest-Telstar) until they were completely dry.

Lyophilized samples were powdered using a Cellcrusher tissue pulverizer (Cellcrusher). The dECM powder obtained was digested with 0.67 mg/mL porcine pepsin (Sigma-Aldrich, Saint Louis, MO, USA) in 0.01 N HCl, in a final 10 mg/mL (dry wt) solution, under a constant stir rate for 48–96 h period at room temperature until the dECM digest was homogeneous, then it was stored at 4 °C.

### 3.2. Human Decellularized Dermal Extracellular Matrix (dECM) Gelation

To neutralize human dECM, 0.01 N NaOH (Sigma 655104) and DMEM 5X (Sigma D5523) were added at 1 tenth and 2.5 tenth of the volume of the dECM digest, respectively. Then, Dulbecco’s Modified Eagle Medium with 4.5 g/L glucose (Biowest, Nuaillé, France), supplemented with 10% fetal bovine serum (Sigma-Aldrich F7524, Saint Louis, MO, USA), 1% penicillin/streptomycin (Lonza), and 1% L-glutamine (Lonza) (DG10) were added to the mixture obtaining the desired concentration for the human dECM hydrogel, 4 mg/mL. All the procedure was performed on ice to avoid quick gelation [60]. The neutralized digests were incubated at 37 °C, 5% CO_2_ for 30–60 min visually following the gelation.

### 3.3. Deoxyribonucliec Acid (DNA) Quantification

Genomic DNA (gDNA) was extracted using DNeasy^®^ Blood &Tissue Kit (Qiagen, Hilden, Germany) following the manufacturer’s protocol. DNA concentration was measured using Take 3 (SynergyTM HT, BioTek, Winooski, VT, USA) and extracted gDNA was run in a 2% agarose gel in TBE 1X buffer with SYBR Safe (Thermo Fisher Scientific, Carlsbad, CA, USA) in an electrophoresis chamber and visualized in a ChemidDC™ XRS + System (BioRad, Los Angeles, CA, USA).

### 3.4. Rheological Characterization

A series of rheological measurements were performed on human dECM hydrogels at 4 mg/mL using a Haake^TM^ Mars^TM^ 40 rheometer equipped with a parallel plate measuring geometry. The diameter of the plate was 25 mm, and the size of the gap was set to 500 μm for all the measurements. The samples were prepared at 0 °C and transferred to the lower plate, which was pre-cooled to 0 °C. After trimming, a thin layer of low viscosity silicon oil was applied to the edge of the sample to prevent evaporation. The temperature of the sample was raised from 0 to 37 °C and it was kept constant at 37 °C for the remainder of the measurement. The temporal evolution of the sample was monitored by performing an oscillatory time sweep. After the equilibration period, a frequency sweep was performed. Both the oscillatory and frequency sweeps were performed within the linear viscoelastic regime at 37 °C. An amplitude sweep was performed, also at 37 °C, after the frequency sweep. Each set of measurement was performed on at least three independent samples of human dECM hydrogel.

### 3.5. Human Decellularized Dermal Extracellular Matrix (dECM) Protein Identification by Liquid Chromatography Mass Spectrometry (LC-MS)

Three human dECM hydrogels from different donors were digested using the iST sample digestion kit from PreOmics (Planegg, Germany) according to the manufacturer’s instructions. This kit includes the steps of lysis, protein denaturation, reduction, alkylation, digestion with LysC and trypsin, and peptide cleanup. Peptide concentration was measured with the Qubit Assay Protein kit on a Qubit 3.0 fluorimeter (Thermo Fisher) following the manufacturer’s instructions. Samples were analyzed in a hybrid trapped ion-mobility quadrupole time-of-flight mass spectrometer (TIMS TOF Flex, Bruker Daltonics, Bremen, Germany) coupled online to an EvoSep ONE liquid chromatograph (EvoSep, Odense C, Denmark). The digested proteins (200 ng) were directly loaded onto the EvoSep ONE chromatograph, and the profiles were acquired using the 60 SPD (samples per day) protocol. Peptides were separated on a C18 column (8 cm × 150 μm, 1.5 μm, PepSep, Bruker) using a linear 21-min gradient and a cycle time of 24 min at a constant flow rate of 1 μL/min. Column temperature was controlled at 40 °C. Data were acquired using the data-dependent acquisition mode with PASEF (parallel accumulation serial fragmentation). MS data were collected over an m/z range from 100 to 1700 and a mobility range of 0.60–1.60 V s/cm^2^. During each MS/MS data collection, each TIMS cycle was 1.1 s and included 1 MS and 10 PASEF MS/MS scans.

Protein identification was carried out with PaSER (2023b, Bruker Scientific LLC, Billerica, MA, USA, http://www.bruker.com, accessed on 9 September 2023) using ProLuCID, DTASelect, and Census. Mass spectra were streamed via the PaSER plugin directly from the acquisition control software to the PaSER workstation via a dedicated LAN connection. Collected data were searched against Uniprot human protein database plus sequences of known contaminants concatenated to a decoy database, in which the sequence for each entry in the original database was reversed using ProLuCID. Twenty ppm precursor tolerance and 30 ppm fragment ion tolerance were used. The search space included all fully-/half-tryptic peptide candidates with 2 missed cleavages. Carbamidomethylation (+57.02146) of cysteine was considered a static modification. TIMScore was appended to raw search results to allow for the use of the peptide Collisional Cross Section (CCS) during the validation process. These results were validated, assembled, and filtered using the DTASelect program with a false discovery rate (FDR) of 0.01; under such filtering conditions, the estimated false discovery rate was below ~1% at the protein level in all analysis.

### 3.6. Biomolecules Quantification within Human Dermal Extracellular Matrix (ECM)

Hydroxyproline content of dECM digest was measured using commercial rat tail type I collagen (Corning™ 354249, Bedford, MA, USA) as control and a standard curve from 0 to 1 µg of hydroxyproline at 560 nm wavelength after processing with the commercial Hydroxyproline Assay kit (Sigma-Aldrich) following the manufacturer’s recommendations. Actual sulphated glycosaminoglycan (sGAG) content of dECM digest was estimated using commercial rat tail type I collagen as negative control and a standard curve from 0 to 10 µg/mL of chondroitin 4 sulfate (Sigma-Aldrich) at 525 nm wavelength by binding with dimethyl methylene blue (DMMB) (Sigma-Aldrich)-GAG complex. The lipid content of dECM and collagen type I hydrogels at 4 mg/mL were quantified at 490 nm after incubating with Oil Red 75% (Sigma-Aldrich) both types of hydrogels for 5 min at room temperature, washing with Mili Q, and extracting Oil Red bound with isopropanol. For the preparation of human dECM hydrogels, the aforementioned steps were followed (see human dECM gelation section in Material and Methods) and for the preparation of collagen type I hydrogels, commercial high concentration rat tail type I collagen, 1 N NaOH at 1:40 ratio (*v*/*v* of collagen volume), DMEM 5X at 1:5 ratio (*v*/*v* of total mixture), and sterile water (if necessary to achieve the final volume) were mixed. The mixture was homogenized on ice avoiding gelation. Once the mixture had a homogenous pink color, the DG10 medium was added. It was homogenized again, using slow pipetting to prevent the formation of bubbles and allowed to polymerize at 37 °C, 5% CO_2_ for 15–20 min. Elastin content of frozen native skin, commercial collagen type I, and dECM digest were quantified at 513 nm wavelength using Fastin™ Elastin Assay Kit (Biocolor, Northern Ireland, UK). Elastin was extracted with 0.25 M oxalic acid three times, heating the samples at 100 °C for 1 h. All the quantification assays were performed in a Take 3 plate reader (Synergy^TM^ HT, BioTek). At least three dECM hydrogels from three different donors were analyzed in each assay.

### 3.7. Immunofluorescence Microscopy

Human dECM hydrogels were performed as described above and fixed in 4% paraformaldehyde for 30 min at room temperature. After fixation, hydrogels were washed with PBS and Tween-20 0.05% in PBS and permeabilized with 0.1% Triton X-100 (Sigma-Aldrich). Next, they were blocked with 10% normal goat serum (Sigma-Aldrich) and washed 3 times with PBS without calcium and magnesium (Lonza) for 15 min. Subsequently, samples were incubated overnight at 4 °C with the following 1:100 diluted primary antibodies in 1.5% normal goat serum: Col4A (SC-59814, Santa Cruz Animal Health, Dallas, TX, USA), Col3A1 (SC-271249, Santa Cruz Animal Health), Fibronectin (SC-8422, Santa Cruz Animal Health), and Col1A (SC-59772, Santa Cruz Animal Health). The next day, samples were washed and incubated for 3 h at room temperature with Alexa Fluor 488 anti-mouse (Thermo Fisher) at 1 µg/mL in 1.5% normal goat serum. All washes were carried out for at least 15 min and under agitation to obtain images with a minimal background signal. Images were visualized at 515–555 nm excitation and 465–495 nm emission wavelength in a Nikon Ti-E coupled to a C1 modular confocal microscope. At least three dECM hydrogels from three different donors were stained.

### 3.8. Scanning Electron Microscopy

Human dECM and type I collagen hydrogels at 4 mg/mL were fixed overnight in 2.5% glutaraldehyde at 4 °C and washed with phosphate buffer (PB) three times. Post-fixation was performed with 2% osmium tetroxide. Next, they were dehydrated in a graded series of alcohol, followed slowly by a critical point of drying in a LEICA EM CPD300 critical point dryer. Hydrogel samples were attached to SEM mounting stubs and coated with Au/Pb (SCD005, Bal-Tec, Santiago de Compostela, Spain). Images were acquired using JSM 6360-LV scanning microscope at 2500 × to visualize the fiber network morphology.

### 3.9. Metabolic Activity and Viability Assays

Human dermal fibroblasts (HDFs) (Gibco) were grown in Dulbecco’s Modified Eagle Medium with 1.0 g/L glucose (Biowest) culture media supplemented with 10% FBS (Sigma F7524), 1% penicillin/streptomycin (Lonza), 1% L-glutamine (Lonza), and 0.5 ng ml^−1^ FGF-2 (Peprotech 100–18B). Human immortalized keratinocytes (HaCaT) (Thermo Fisher Scientific) were grown in Dulbecco’s Modified Eagle Medium with 4.5 g/L glucose (Biowest) culture media supplemented with 10% FBS (Sigma F7524), 1% penicillin/streptomycin (Lonza), and 1% L-glutamine (Lonza). Both cell lines were cultivated at 37 °C, 5% CO_2_/95% air atmosphere and media were refreshed every other day. After reaching 80% of confluence, cells were passaged.

HDFs at 1.6 × 10^5^ cells/mL density were embedded within dECM hydrogels or collagen type I hydrogels as control, both at 4 mg/mL. For the preparation of both hydrogels, the aforementioned steps were followed (see Human dECM Gelation and Biomolecules quantification within human dermal ECM sections in Material and Methods). The fibroblast suspension was added in the last step along the DG10 medium, homogenized, and then 50 µL of the mixture was added to each well of a 96-well plate and allowed to polymerize. After polymerization, the medium was added to the surface of the hydrogels. HaCaT cells were seeded on top of both types of hydrogels (without embedded fibroblast) at 10^6^ keratinocytes/well density. Experiment media were refreshed every other day. Cell metabolic activity was quantified by 3-(4,5-dimethylthiazol-2-yl)-2,5-diphenyltetrazolium bromide, MTT (Sigma-Aldrich). After 48 h and 1 week, each sample was exposed to 150 μL of MTT for 4 h at 37 °C, and then dissolving the crystals in 150 μL of isopropyl alcohol for 3 h at 37 °C. Absorbance was quantified in a Synergy^TM^ HT, BioTek plate reader, at 570 nm wavelength with 690 nm wavelength as reference. At least three independent experiments with three replicates each were performed. Embedded HDF in human dermal ECM and collagen type I hydrogels were also stained with 5 μg/mL of Calcein-AM (CAM) (Thermo Fisher Scientific, Life Technologies, C1430, Eugene, OR, USA) and 4 μL/mL of propidium iodide (PI) (Sigma P4170) in PBS for 30 min at 37 °C after one week of culture. Cells were visualized under a DMi8 Thunder microscope (Leica, L’Hospitalet de Llobregat, Spain).

### 3.10. Cell Tracking of Dermal Fibroblast Embedded in Human Dermal Extracellular Matrix (ECM) Hydrogels within a Microfluidic Device

Customized microfluidic devices based on cyclic olefin polymers (COPs) were designed and fabricated by Beonchip company. The microdevice consisted of two independent open culture wells communicated through a 1 μm porous polyethylene terephthalate (PET) membrane with a 150 µm thickness intermediate chamber in each culture well, which in turn was communicated at the bottom with another membrane with its respective microfluidic channel.

HDFs and HaCaT cells were labeled with Vybrant DiO and DiI dyes, respectively, by incubating with 5 μL dye/10^6^ cells at 37 °C for 15 min, followed by washing. Labeled Vybrant DiO HDFs at 1.6 × 10^5^ cells/mL density were embedded in 4 mg/mL human dermal ECM hydrogels or collagen hydrogels as a control, as previously described, and pushed the embedded fibroblasts within the human dECM or collagen hydrogel in the intermediate chamber of the microfluidic device, then sealed after the hydrogels were within, incubating at 37 °C for 30 min upon gelation. Afterwards, the preheated culture medium was perfused through the microfluidic channels incubating at 37 °C. The next day, labeled DiI 10^6^ HaCaT cells were seeded on the open well immersed in a culture medium for 4 days, incubating without flow for the first 24 h, and with a constant passive flow generated by tilt movement in a rocker during the rest of the experiment. The medium in both channels and open well was refreshed daily. Four days after seeding the keratinocytes, the medium was removed, creating an air–liquid interface (ALI) for at least 12 days (Figure 3G). The microdevice allowed for feeding the cultured cells from the bottom microfluidic channels, where the culture medium can be provided with a passive flow. Moreover, the open chamber where keratinocytes were grown allowed for generating an air–liquid interface, which is required for the differentiation of keratinocytes to form the epidermis.

Labeled cells were visualized under a DMi8 Thunder microscope (Leica) at 500–550 nm excitation and 450–490 nm emission wavelength for DiO, and 541–551 nm excitation and 565–605 nm emission wavelength for DiI, generating Z-stack images overtime. Fibroblast migration within collagen and dECM hydrogels was analyzed by quantifying fluorescence intensities of DiO and DiI emission through Z-stacks with ImageJ-Fiji 1.53q [61]. Three independent experiments were performed.

### 3.11. Statistical Analysis

Significant differences in human dECM biomolecules quantifications and cellular metabolic activity were determined by the unpaired t-Student test, with normal distribution assessed by the Shapiro-Wilk test. Both tests were conducted using the statistical software GraphPad, version 8.02 (GraphPad Inc., San Diego, CA, USA). Data are expressed as a mean ± standard deviation. All experiments were performed at least three times, and *p*-values from *p* < 0.05 to *p* < 0.0001 were considered statistically significant.

## 4. Conclusions

In this paper, we have confirmed the potential of human dECM hydrogels extracted from adult cadaveric human skin as a close natural niche for dermal fibroblast to be used as scaffolds in advanced skin aging models. Human dECM hydrogels reveal a porous fibrillar architecture, with a biomolecular composition closer to the dermis than collagen hydrogels and a mechanical behavior similar to the reported commercial materials such as collagen type I. Within human dECM, the behavior of the fibroblast resembles natural adult skin, in contrast to collagen type I hydrogels where cells provoke the shrinkage of the biomaterial, attributed to the controlled proliferation of fibroblasts within the dECM hydrogels. Moreover, fibroblasts within adult human dECM show a closer distribution to adult skin than fibroblasts embedded in collagen type I hydrogels. In conclusion, the described human dECM hydrogels preserve the essential components of native adult dermis and provide a biocompatible and realistic microenvironment for advanced skin aging models. The findings of the current study have significant implications for the development of new in vitro skin aging models, potentially improving the quality of testing platforms for drug testing or in dermatopathological research.

## Data Availability

Data available on request from the authors. The data that support the findings of this study are available from the corresponding author [J.C.] upon reasonable request.

## Supplementary Materials

The following supporting information can be downloaded at: https://www.mdpi.com/article/10.3390/ijms25074020/s1.

## References

[1] van den Broek L.J., Bergers L.I.J.C., Reijnders C.M.A., Gibbs S. (2017). Progress and Future Prospectives in Skin-on-Chip Development with Emphasis on the use of Different Cell Types and Technical Challenges. Stem Cell Rev. Rep..

[2] Monteiro-Riviere N.A. (2010). Structure and Function of Skin. Dermal Absorpt. Models Toxicol. Pharmacol..

[3] Pissarenko A., Meyers M.A. (2020). The materials science of skin: Analysis, characterization, and modeling. Prog. Mater. Sci..

[4] Fernandez-Carro E., Angenent M., Gracia-Cazaña T., Gilaberte Y., Alcaine C., Ciriza J. (2022). Modeling an Optimal 3D Skin-on-Chip within Microfluidic Devices for Pharmacological Studies. Pharmaceutics.

[5] Freytes D.O., Martin J., Velankar S.S., Lee A.S., Badylak S.F. (2008). Preparation and rheological characterization of a gel form of the porcine urinary bladder matrix. Biomaterials.

[6] Risueño I., Valencia L., Jorcano J.L., Velasco D. (2021). Skin-on-a-chip models: General overview and future perspectives. APL Bioeng..

[7] Beißner N., Albero A.B., Füller J., Kellner T., Lauterboeck L., Liang J., Böl M., Glasmacher B., Müller-Goymann C.C., Reichl S. (2018). Improved in vitro models for preclinical drug and formulation screening focusing on 2D and 3D skin and cornea constructs. Eur. J. Pharm. Biopharm..

[8] Lee S., Jin S.-P., Kim Y.K., Sung G.Y., Chung J.H., Sung J.H. (2017). Construction of 3D multicellular microfluidic chip for an in vitro skin model. Biomed. Microdevices.

[9] Hwang S.J., Kim S.H., Seo W.-Y., Jeong Y., Shin M.C., Ryu D., Lee S.B., Choi Y.J., Kim K. (2021). Effects of human collagen α-1 type I-derived proteins on collagen synthesis and elastin production in human dermal fibroblasts. BMB Rep..

[10] Woodley J.P., Lambert D.W., Asencio I.O. (2022). Understanding Fibroblast Behavior in 3D Biomaterials. Tissue Eng. Part. B Rev..

[11] Mithieux S.M., Weiss A.S. (2005). Elastin. Adv. Protein Chem..

[12] Adamo C.S., Zuk A.V., Sengle G. (2020). The fibrillin microfibril/elastic fibre network: A critical extracellular supramolecular scaffold to balance skin homoeostasis. Exp. Dermatol..

[13] Smith L.T., Holbrook K.A., Madri J.A. (1986). Collagen types I, III, and V in human embryonic and fetal skin. Am. J. Anat..

[14] Xue M., Jackson C.J. (2015). Extracellular Matrix Reorganization During Wound Healing and Its Impact on Abnormal Scarring. Adv. Wound Care.

[15] Huang J., Heng S., Zhang W., Liu Y., Xia T., Ji C., Zhang L.-J. (2022). Dermal extracellular matrix molecules in skin development, homeostasis, wound regeneration and diseases. Semin. Cell Dev. Biol..

[16] Uitto J., Olsen D.R., Fazio M.J. (1989). Extracellular Matrix of the Skin: 50 Years of Progress. J. Investig. Dermatol..

[17] Pfisterer K., Shaw L.E., Symmank D., Weninger W. (2021). The Extracellular Matrix in Skin Inflammation and Infection. Front. Cell Dev. Biol..

[18] Mouw J.K., Ou G., Weaver V.M. (2014). Extracellular matrix assembly: A multiscale deconstruction. Nat. Rev. Mol. Cell Biol..

[19] Tracy L.E., Minasian R.A., Caterson E. (2016). Extracellular Matrix and Dermal Fibroblast Function in the Healing Wound. Adv. Wound Care.

[20] Penc S.F., Pomahac B., Winkler T., Dorschner R.A., Eriksson E., Herndon M., Gallo R.L. (1998). Dermatan Sulfate Released after Injury Is a Potent Promoter of Fibroblast Growth Factor-2 Function. J. Biol. Chem..

[21] Zhou H.-M., Wang J., Elliott C., Wen W., Hamilton D.W., Conway S.J. (2010). Spatiotemporal expression of periostin during skin development and incisional wound healing: Lessons for human fibrotic scar formation. J. Cell Commun. Signal..

[22] Järvinen T.A.H., Prince S. (2015). Decorin: A Growth Factor Antagonist for Tumor Growth Inhibition. BioMed Res. Int..

[23] Takeda U., Utani A., Wu J., Shinkai H., Adachi E., Koseki H., Taniguchi M., Matsumoto T., Ohashi T., Sato M. (2002). Targeted Disruption of Dermatopontin Causes Abnormal Collagen Fibrillogenesis. J. Investig. Dermatol..

[24] Saldin L.T., Cramer M.C., Velankar S.S., White L.J., Badylak S.F. (2017). Extracellular matrix hydrogels from decellularized tissues: Structure and function. Acta Biomater..

[25] Wolf M.T., Daly K.A., Brennan-Pierce E.P., Johnson S.A., Carruthers C.A., D’Amore A., Nagarkar S.P., Velankar S.S., Badylak S.F. (2012). A hydrogel derived from decellularized dermal extracellular matrix. Biomaterials.

[26] Sarmin A.M., El Moussaid N., Suntornnond R., Tyler E.J., Kim Y.-H., Di Cio S., Megone W.V., Pearce O., Gautrot J.E., Dawson J. (2022). Multi-Scale Analysis of the Composition, Structure, and Function of Decellularized Extracellular Matrix for Human Skin and Wound Healing Models. Biomolecules.

[27] Khiao In M., Richardson K.C., Loewa A., Hedtrich S., Kaessmeyer S., Plendl J. (2019). Histological and functional comparisons of four anatomical regions of porcine skin with human abdominal skin. Anat. Histol. Embryol..

[28] Cicuéndez M., Casarrubios L., Feito M.J., Madarieta I., Garcia-Urkia N., Murua O., Olalde B., Briz N., Diez-Orejas R., Portolés M.T. (2021). Effects of Human and Porcine Adipose Extracellular Matrices Decellularized by Enzymatic or Chemical Methods on Macrophage Polarization and Immunocompetence. Int. J. Mol. Sci..

[29] Liao P., Wang Z. (2019). Thiel-embalming technique: Investigation of possible modification in embalming tissue as evaluation model for radiofrequency ablation. J. Biomed. Res..

[30] Yadav M., Chaudhary P.P., D’souza B.N., Spathies J., Myles I.A. (2022). Impact of Skin Tissue Collection Method on Downstream MALDI-Imaging. Metabolites.

[31] Crosado B., Löffler S., Ondruschka B., Zhang M., Zwirner J., Hammer N. (2019). Phenoxyethanol-Based Embalming for Anatomy Teaching: An 18 Years’ Experience with Crosado Embalming at the University of Otago in New Zealand. Anat. Sci. Educ..

[32] Rohn S., Rawel H.M., Kroll J. (2002). Inhibitory Effects of Plant Phenols on the Activity of Selected Enzymes. J. Agric. Food Chem..

[33] Abaci A., Guvendiren M. (2020). Designing Decellularized Extracellular Matrix-Based Bioinks for 3D Bioprinting. Adv. Health Mater..

[34] Crapo P.M., Gilbert T.W., Badylak S.F. (2011). An overview of tissue and whole organ decellularization processes. Biomaterials.

[35] White L.J., Taylor A.J., Faulk D.M., Keane T.J., Saldin L.T., Reing J.E., Swinehart I.T., Turner N.J., Ratner B.D., Badylak S.F. (2016). The impact of detergents on the tissue decellularization process: A ToF-SIMS study. Acta Biomater..

[36] Lamers E., Van Kempen T.H.S., Baaijens F.P.T., Peters G.W.M., Oomens C.W.J. (2013). Large amplitude oscillatory shear properties of human skin. J. Mech. Behav. Biomed. Mater..

[37] Silver F.H., Freeman J.W., DeVore D. (2001). Viscoelastic properties of human skin and processed dermis. Ski. Res. Technol..

[38] Silver F.H., Siperko L.M., Seehra G.P. (2003). Mechanobiology of force transduction in dermal tissue. Ski. Res. Technol..

[39] Pailler-Mattei C., Debret R., Vargiolu R., Sommer P., Zahouani H. (2013). In vivo skin biophysical behaviour and surface topography as a function of ageing. J. Mech. Behav. Biomed. Mater..

[40] Boyer G., Laquièze L., Le Bot A., Laquièze S., Zahouani H. (2009). Dynamic indentation on human skin in vivo: Ageing effects. Ski. Res. Technol..

[41] Jachowicz J., McMullen R., Prettypaul D. (2007). Indentometric analysis of in vivo skin and comparison with artificial skin models. Ski. Res. Technol..

[42] Castro-Abril H., Heras J., del Barrio J., Paz L., Alcaine C., Aliácar M.P., Garzón-Alvarado D., Doblaré M., Ochoa I. (2023). The Role of Mechanical Properties and Structure of Type I Collagen Hydrogels on Colorectal Cancer Cell Migration. Macromol. Biosci..

[43] Sackett S.D., Tremmel D.M., Ma F., Feeney A.K., Maguire R.M., Brown M.E., Zhou Y., Li X., O’brien C., Li L. (2018). Extracellular matrix scaffold and hydrogel derived from decellularized and delipidized human pancreas. Sci. Rep..

[44] Uitto J., Perejda A.J., Abergel R.P., Chu M.L., Ramirez F. (1985). Altered steady-state ratio of type I/III procollagen mRNAs correlates with selectively increased type I procollagen biosynthesis in cultured keloid fibroblasts. Proc. Natl. Acad. Sci. USA.

[45] Bonnans C., Chou J., Werb Z. (2014). Remodelling the extracellular matrix in development and disease. Nat. Rev. Mol. Cell Biol..

[46] Okamoto O., Fujiwara S. (2006). Dermatopontin, a Novel Player in the Biology of the Extracellular Matrix. Connect. Tissue Res..

[47] Lewandowska K., Choi H.U., Rosenberg L.C., Sasse J., Ame P.J.N., Culp L.A. (1991). Extracellular matrix adhesion-promoting activities of a dermatan sulfate proteoglycan-associated protein (22K) from bovine fetal skin. J. Cell Sci..

[48] Langton A.K., Sherratt M.J., Griffiths C.E.M., Watson R.E.B. (2010). Review Article: A new wrinkle on old skin: The role of elastic fibres in skin ageing. Int. J. Cosmet. Sci..

[49] Qin Z., Fisher G.J., Voorhees J.J., Quan T. (2018). Actin cytoskeleton assembly regulates collagen production via TGF-β type II receptor in human skin fibroblasts. J. Cell. Mol. Med..

[50] Theocharidis G., Connelly J.T. (2017). Minor collagens of the skin with not so minor functions. J. Anat..

[51] Kim B.S., Kwon Y.W., Kong J.-S., Park G.T., Gao G., Han W., Kim M.-B., Lee H., Kim J.H., Cho D.-W. (2018). 3D cell printing of in vitro stabilized skin model and in vivo pre-vascularized skin patch using tissue-specific extracellular matrix bioink: A step towards advanced skin tissue engineering. Biomaterials.

[52] Duval K., Grover H., Han L.-H., Mou Y., Pegoraro A.F., Fredberg J., Chen Z. (2017). Modeling Physiological Events in 2D vs. 3D Cell Culture. Physiology.

[53] Law A.M.K., de la Fuente L.R., Grundy T.J., Fang G., Valdes-Mora F., Gallego-Ortega D. (2021). Advancements in 3D Cell Culture Systems for Personalizing Anti-Cancer Therapies. Front. Oncol..

[54] Fang G., Chen Y., Lu H., Jin D. (2023). Advances in Spheroids and Organoids on a Chip. Adv. Funct. Mater..

[55] Yue B. (2014). Biology of the Extracellular Matrix. Eur. J. Gastroenterol. Hepatol..

[56] Ingber D.E. (2008). Tensegrity-based mechanosensing from macro to micro. Prog. Biophys. Mol. Biol..

[57] Kim K., Kim J., Kim H., Sung G.Y. (2021). Effect of α-Lipoic Acid on the Development of Human Skin Equivalents Using a Pumpless Skin-on-a-Chip Model. Int. J. Mol. Sci..

[58] Kim J., Kim K., Sung G.Y. (2020). Coenzyme Q10 Efficacy Test for Human Skin Equivalents Using a Pumpless Skin-On-A-Chip System. Int. J. Mol. Sci..

[59] Sorrell J.M., Caplan A.I. (2004). Fibroblast heterogeneity: More than skin deep. J. Cell Sci..

[60] Ozpinar E.W., Frey A.L., Arthur G.K., Mora-Navarro C., Biehl A., Snider D.B., Cruse G., Freytes D.O. (2021). Dermal Extracellular Matrix-Derived Hydrogels as an In Vitro Substrate to Study Mast Cell Maturation. Tissue Eng. Part A.

[61] Schneider C.A., Rasband W.S., Eliceiri K.W. (2012). NIH Image to ImageJ: 25 Years of image analysis. Nat. Methods.

