# Supplementary material for: Human Dermal Decellularized ECM Hydrogels as Scaffolds for 3D In Vitro Skin Aging Models
Source: Int J Mol Sci. 2024 Apr 4;25(7):4020. doi: 10.3390/ijms25074020 (PMC11011913; doi:10.3390/ijms25074020)
Supplement: Supplementary file 1 [file ijms-25-04020-s001.zip › ijms-2927773-supplementary.pdf]

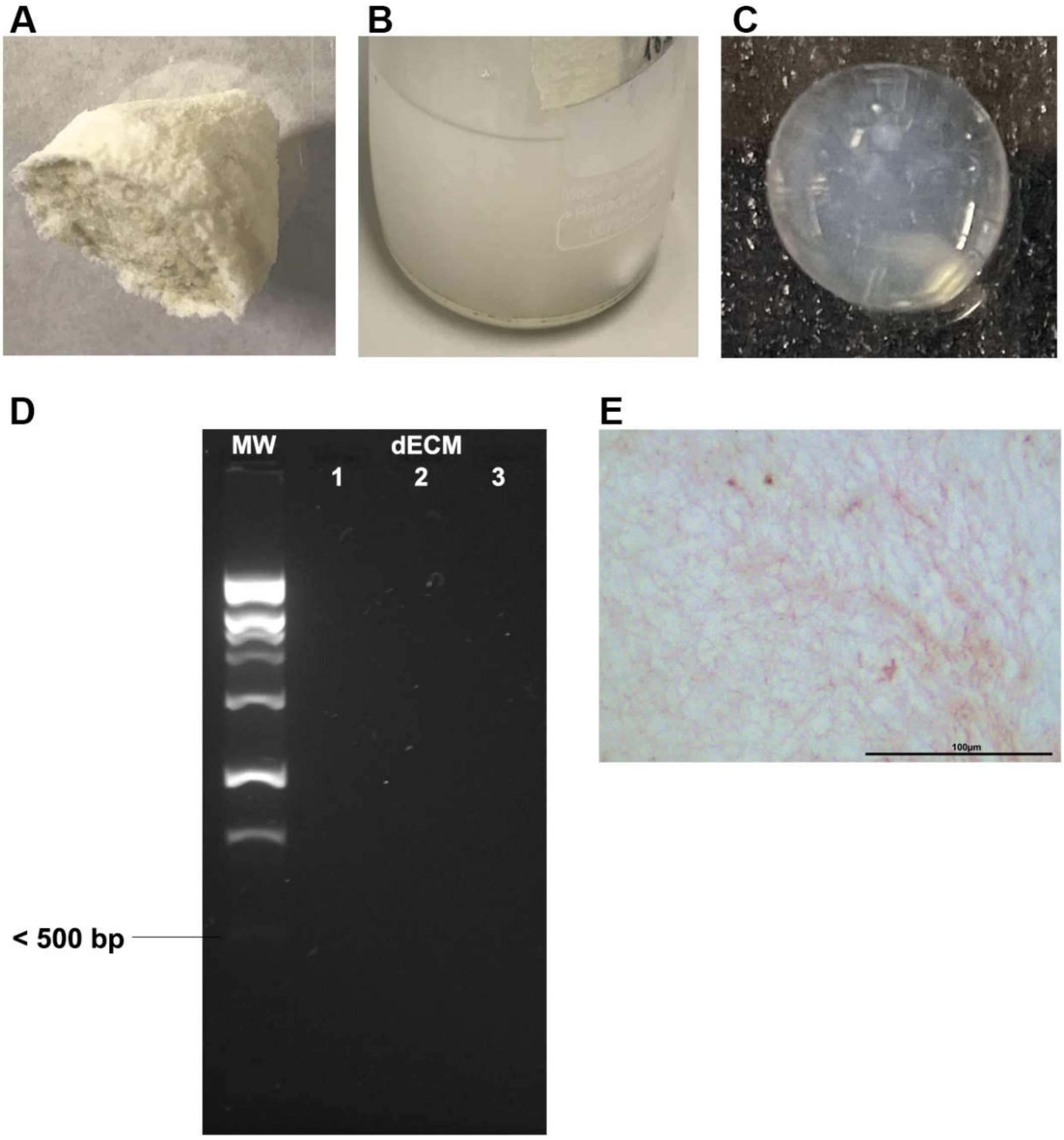

Figure S1. (A) Lyophilized skin after extraction with sponge-like morphology, (B) human dECM solution after pepsin digestion and (C) human dECM hydrogel at 4 mg/mL. (D) 2% agarose gel electrophoresis showing absence of residual DNA content and (E) haematoxylin-eosin stain showing the lack of cellular nuclei in human dECM.

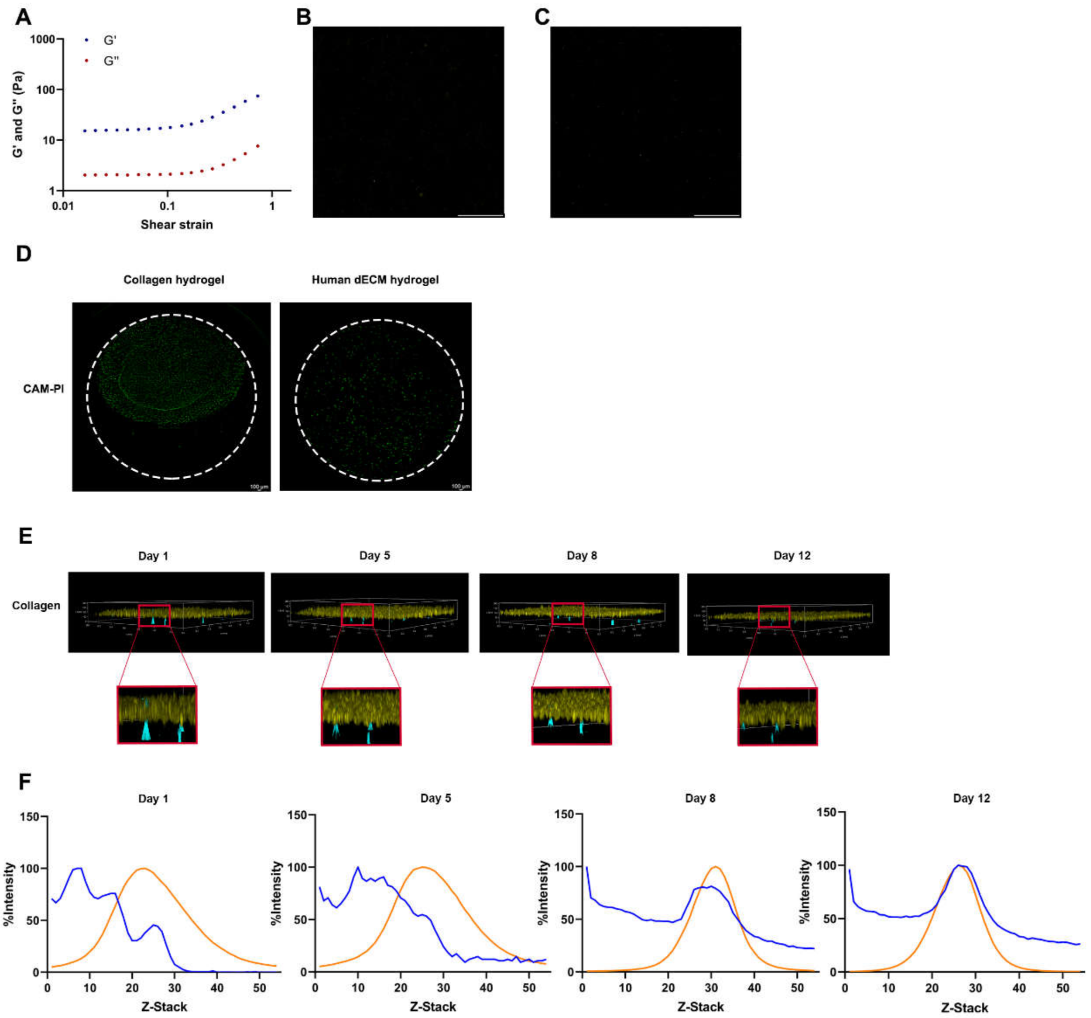

Figure S2. (A) Amplitude sweep of human dECM hydrogel. (B) Negative control of immunofluorescence for collagen type I, type III, type IV and fibronectin, and (C) immunofluorescence of collagen type IV. (D) Collagen hydrogel shrinkage by dermal fibroblast and lack of contraction in dECM hydrogels where circle indicates the original perimeter of the hydrogel. (E) Keratinocytes (yellow) on top of collagen type I hydrogels and fibroblast embedded in collagen type I hydrogel (blue) tracking overtime within a skin-on-chip device. (F) Analysis of cell migration quantifying the fluorescence intensity of fibroblasts (blue) embedded in collagen type I hydrogel and keratinocytes on top of hydrogel (orange) over time within skin on chip deviceNote: Immunofluorescence scale bar: 50 µm. CAM-PI: Live/Dead staining.

Table S1. Protein identification by liquid chromatography mass spectrometry from three independent human hydrogel dECM.

Accesion number	Gene name	Seq count	EMPAI	Description
		dECM 1		
		Collagens		
P02452	COL1A1	218	1.93	Collagen alpha-1(I) chain OS=Homo sapiens OX=9606 GN=COL1A1 PE=1 SV=6
P08123	COL1A2	172	1.71	Collagen alpha-2(I) chain OS=Homo sapiens OX=9606 GN=COL1A2 PE=1 SV=7
P02461	COL3A1	80	0.54	Collagen alpha-1(III) chain OS=Homo sapiens OX=9606 GN=COL3A1 PE=1 SV=4
P02458	COL2A1	53	0.32	Collagen alpha-1(II) chain OS=Homo sapiens OX=9606 GN=COL2A1 PE=1 SV=3
P05997	COL5A2	13	0.17	Collagen alpha-2(V) chain OS=Homo sapiens OX=9606 GN=COL5A2 PE=1 SV=3
P20908	COL5A1	20	0.16	Collagen alpha-1(V) chain OS=Homo sapiens OX=9606 GN=COL5A1 PE=1 SV=3
P12111	COL6A3	12	0.12	Collagen alpha-3(VI) chain OS=Homo sapiens OX=9606 GN=COL6A3 PE=1 SV=5
		Keratins		
P04264	KRT1	36	0.85	Keratin, type II cytoskeletal 1 OS=Homo sapiens OX=9606 GN=KRT1 PE=1 SV=6
P04259	KRT6B	12	0.31	Keratin, type II cytoskeletal 6B OS=Homo sapiens OX=9606 GN=KRT6B PE=1 SV=5
Q5XKE5	KRT79	11	0.28	Keratin, type II cytoskeletal 79 OS=Homo sapiens OX=9606 GN=KRT79 PE=1 SV=2
Q14CN4	KRT72	8	0.21	Keratin, type II cytoskeletal 72 OS=Homo sapiens OX=9606 GN=KRT72 PE=1 SV=2
O95678	KRT75	13	0.21	Keratin, type II cytoskeletal 75 OS=Homo sapiens OX=9606 GN=KRT75 PE=1 SV=2
P08729	KRT7	7	0.19	Keratin, type II cytoskeletal 7 OS=Homo sapiens OX=9606 GN=KRT7 PE=1 SV=5
Q86Y46	KRT73	4	0.17	Keratin, type II cytoskeletal 73 OS=Homo sapiens OX=9606 GN=KRT73 PE=1 SV=1
Q7Z794	KRT77	7	0.15	Keratin, type II cytoskeletal 1b OS=Homo sapiens OX=9606 GN=KRT77 PE=1 SV=3
Q3SY84	KRT71	7	0.14	Keratin, type II cytoskeletal 71 OS=Homo sapiens OX=9606 GN=KRT71 PE=1 SV=3
Q7RTS7	KRT74	4	0.13	Keratin, type II cytoskeletal 74 OS=Homo sapiens OX=9606 GN=KRT74 PE=1 SV=2
		Other components of the dECM		
P60709	ACTB	14	0.57	Actin, cytoplasmic 1 OS=Homo sapiens OX=9606 GN=ACTB PE=1 SV=1
P63261	ACTG1		0.57	Actin, cytoplasmic 2 OS=Homo sapiens OX=9606 GN=ACTG1 PE=1 SV=1
Q07507	DPT	9	0.36	Dermatopontin OS=Homo sapiens OX=9606 GN=DPT PE=1 SV=2
P15502	ELN	6	0.11	Elastin OS=Homo sapiens OX=9606 GN=ELN PE=1 SV=4
P35555	FBN1	15	0.09	Fibrillin-1 OS=Homo sapiens OX=9606 GN=FBN1 PE=1 SV=4
		dECM 2		
**Accesion number**	**Gene name**	**Seq count**	**EMPAI**	**Description**
Collagens				

P02452	COL1A1	232	1.90	Collagen alpha-1(I) chain OS=Homo sapiens OX=9606 GN=COL1A1 PE=1 SV=6
P08123	COL1A2	178	1.70	Collagen alpha-2(I) chain OS=Homo sapiens OX=9606 GN=COL1A2 PE=1 SV=7
P02461	COL3A1	75	0.54	Collagen alpha-1(III) chain OS=Homo sapiens OX=9606 GN=COL3A1 PE=1 SV=4
P02458	COL2A1	56	0.46	Collagen alpha-1(II) chain OS=Homo sapiens OX=9606 GN=COL2A1 PE=1 SV=3
P12109	COL6A1	14	0.29	Collagen alpha-1(VI) chain OS=Homo sapiens OX=9606 GN=COL6A1 PE=1 SV=3
P20908	COL5A1	23	0.26	Collagen alpha-1(V) chain OS=Homo sapiens OX=9606 GN=COL5A1 PE=1 SV=3
P25940	COL5A3	13	0.24	Collagen alpha-3(V) chain OS=Homo sapiens OX=9606 GN=COL5A3 PE=1 SV=3
P12111	COL6A3	24	0.15	Collagen alpha-3(VI) chain OS=Homo sapiens OX=9606 GN=COL6A3 PE=1 SV=5
P05997	COL5A2	14	0.13	Collagen alpha-2(V) chain OS=Homo sapiens OX=9606 GN=COL5A2 PE=1 SV=3
P12110	COL6A2	5	0.10	Collagen alpha-2(VI) chain OS=Homo sapiens OX=9606 GN=COL6A2 PE=1 SV=4
Keratins				
P04264	KRT1	40	0.77	Keratin, type II cytoskeletal 1 OS=Homo sapiens OX=9606 GN=KRT1 PE=1 SV=6
P04259	KRT6B	14	0.61	Keratin, type II cytoskeletal 6B OS=Homo sapiens OX=9606 GN=KRT6B PE=1 SV=5
Q5XKE5	KRT79	14	0.46	Keratin, type II cytoskeletal 79 OS=Homo sapiens OX=9606 GN=KRT79 PE=1 SV=2
O95678	KRT75	12	0.18	Keratin, type II cytoskeletal 75 OS=Homo sapiens OX=9606 GN=KRT75 PE=1 SV=2
		Other components of the dECM		
P35555	FBN1	13	0.07	Fibrillin-1 OS=Homo sapiens OX=9606 GN=FBN1 PE=1 SV=4

dECM 3

**Accesion number**	**Gene name**	**Seq count**	**EMPAI**	**Description**
Collagens				
P02452	COL1A1	248	2.10	Collagen alpha-1(I) chain OS=Homo sapiens OX=9606 GN=COL1A1 PE=1 SV=6
P08123	COL1A2	184	1.76;	Collagen alpha-2(I) chain OS=Homo sapiens OX=9606 GN=COL1A2 PE=1 SV=7
P02461	COL3A1	87	0.56	Collagen alpha-1(III) chain OS=Homo sapiens OX=9606 GN=COL3A1 PE=1 SV=4
P02458	COL2A1	52	0.34	Collagen alpha-1(II) chain OS=Homo sapiens OX=9606 GN=COL2A1 PE=1 SV=3
P12109	COL6A1	10	0.25	Collagen alpha-1(VI) chain OS=Homo sapiens OX=9606 GN=COL6A1 PE=1 SV=3
P05997	COL5A2	14	0.18	Collagen alpha-2(V) chain OS=Homo sapiens OX=9606 GN=COL5A2 PE=1 SV=3
P12111	COL6A3	30	0.16	Collagen alpha-3(VI) chain OS=Homo sapiens OX=9606 GN=COL6A3 PE=1 SV=5
P20908	COL5A1	22	0.16	Collagen alpha-1(V) chain OS=Homo sapiens OX=9606 GN=COL5A1 PE=1 SV=3
P25940	COL5A3	8	0.11	Collagen alpha-3(V) chain OS=Homo sapiens OX=9606 GN=COL5A3 PE=1 SV=3
Keratins				

Q7Z794	KRT77	9	0.41	Keratin, type II cytoskeletal 1b OS=Homo sapiens OX=9606 GN=KRT77 PE=1 SV=3
Q5XKE5	KRT79	11	0.36	Keratin, type II cytoskeletal 79 OS=Homo sapiens OX=9606 GN=KRT79 PE=1 SV=2
P04264	KRT1	14	0.31	Keratin, type II cytoskeletal 1 OS=Homo sapiens OX=9606 GN=KRT1 PE=1 SV=6
Q6KB66	KRT80	6	0.30	Keratin, type II cytoskeletal 80 OS=Homo sapiens OX=9606 GN=KRT80 PE=1 SV=2
O95678	KRT75	12	0.28	Keratin, type II cytoskeletal 75 OS=Homo sapiens OX=9606 GN=KRT75 PE=1 SV=2
P04259	KRT6B	10	0.24	Keratin, type II cytoskeletal 6B OS=Homo sapiens OX=9606 GN=KRT6B PE=1 SV=5
Q9NSB2	KRT84	4	0.19	Keratin, type II cuticular Hb4 OS=Homo sapiens OX=9606 GN=KRT84 PE=2 SV=2
Q86Y46	KRT73	3	0.16	Keratin, type II cytoskeletal 73 OS=Homo sapiens OX=9606 GN=KRT73 PE=1 SV=1
Q14CN4	KRT72	4	0.15	Keratin, type II cytoskeletal 72 OS=Homo sapiens OX=9606 GN=KRT72 PE=1 SV=2
Q3SY84	KRT71	5	0.13	Keratin, type II cytoskeletal 71 OS=Homo sapiens OX=9606 GN=KRT71 PE=1 SV=3
		Other components of the dECM		
P07585	DCN	5	0.39	Decorin OS=Homo sapiens OX=9606 GN=DCN PE=1 SV=1
P15502	ELN	16	0.21	Elastin OS=Homo sapiens OX=9606 GN=ELN PE=1 SV=4
